# Image-Based Network Analysis of DNp73 Expression by Immunohistochemistry in Rectal Cancer Patients

**DOI:** 10.3389/fphys.2019.01551

**Published:** 2020-01-08

**Authors:** Tuan D. Pham, Chuanwen Fan, Daniella Pfeifer, Hong Zhang, Xiao-Feng Sun

**Affiliations:** ^1^Department of Biomedical Engineering, Linkoping University, Linkoping, Sweden; ^2^The Center for Artificial Intelligence, Prince Mohammad Bin Fahd University, Al Khobar, Saudi Arabia; ^3^Department of Oncology, Clinical and Experimental Medicine, Linkoping University, Linkoping, Sweden; ^4^Institute of Digestive Surgery, West China Hospital, Sichuan University, Chengdu, China; ^5^Department of Medical Sciences, Orebro University, Orebro, Sweden

**Keywords:** fuzzy weighted recurrence networks, network properties, multi-channel images, DNp73, immunohistochemistry, predictive biomarker, rectal cancer, survival outcome

## Abstract

**Background:** Rectal cancer is a disease characterized with tumor heterogeneity. The combination of surgery, radiotherapy, and chemotherapy can reduce the risk of local recurrence. However, there is a significant difference in the response to radiotherapy among rectal cancer patients even they have the same tumor stage. Despite rapid advances in knowledge of cellular functions affecting radiosensitivity, there is still a lack of predictive factors for local recurrence and normal tissue damage. The tumor protein DNp73 is thought as a biomarker in colorectal cancer, but its clinical significance is still not sufficiently investigated, mainly due to the limitation of human-based pathology analysis. In this study, we investigated the predictive value of DNp73 in patients with rectal adenocarcinoma using image-based network analysis.

**Methods:** The fuzzy weighted recurrence network of time series was extended to handle multi-channel image data, and applied to the analysis of immunohistochemistry images of DNp73 expression obtained from a cohort of 25 rectal cancer patients who underwent radiotherapy before surgery. Two mathematical weighted network properties, which are the clustering coefficient and characteristic path length, were computed for the image-based networks of the primary tumor (obtained after operation) and biopsy (obtained before operation) of each cancer patient.

**Results:** The ratios of two weighted recurrence network properties of the primary tumors to biopsies reveal the correlation of DNp73 expression and long survival time, and discover the non-effective radiotherapy to a cohort of rectal cancer patients who had short survival time.

**Conclusion:** Our work contributes to the elucidation of the predictive value of DNp73 expression in rectal cancer patients who were given preoperative radiotherapy. Mathematical properties of fuzzy weighted recurrence networks of immunohistochemistry images are not only able to show the predictive factor of DNp73 expression in the patients, but also reveal the identification of non-effective application of radiotherapy to those who had poor overall survival outcome.

## 1. Introduction

Colorectal cancer (CRC) is the third most common cancer in the world (Arnold et al., 2017). There are around 6,500 new diagnosed cases of CRC yearly among the population of Sweden. The causes for CRC are considered to be associated with gene mutations, gene variants, and changed expression of proteins. The combination of surgery and radio-chemotherapy is the most beneficial regimens in current treatment of advanced rectal cancer. Preoperative radiotherapy (RT) is often given to rectal cancer patients as a complement to surgery to improve treatment outcome. However, tumor recurrence plays a major cause of death for progressive rectal patients after surgery. As a result, a significant proportion of patients did not benefit from preoperative RT (Fan et al., 2013).

It remains up to date that the clinical testing of specific mutations in KRAS, BRAF, RAS, and RAF genes along with mismatch repair gene deficiency assists either as prognostic or predictive biomarkers in CRC (Sinicrope et al., 2016; Zarkavelis et al., 2017). Other methods for identifying biomarkers in the treatment of CRC include molecular subtype classification (Cuyle and Prenen, 2017), identifying molecular signatures at protein and RNA levels by microarray analysis (Rahman et al., 2019), protein identification in cell proliferation and new blood vessels (Chatterjee et al., 2019), changes in the amounts of certain proteins (Letellier et al., 2017), and proteomic strategies (Lee et al., 2018). A recent review of methods for discovering prognostic and predictive biomarkers in CRC for personalized therapy can be found in Patel et al. (2019).

The role of predictive biomarkers is known to be essential for the field of radiation oncology (Yaromina et al., 2012). While most efforts aim to improve cancer treatment with respect to physical conditions and technology, such as precision in treatment plans and dose administration (Sonke and Belderbos, 2010), the inclusion of patient-specific biological characteristics into cancer treatment decision would be very useful for personalized treatment. However, such important biological information of individual patients is not well explored. To achieve this purpose, predictive biomarkers are needed to guide radiation oncologists to determine optimal dose prescription, select patient-specific schemes, and treatments for individual cancer patients (Yaromina et al., 2012).

However, it is a big challenge to find predictive biomarkers that can select patients who can benefit from RT, although our and other groups have spent much effort to identify potential predictors for the RT response (Ryan et al., 2016; Ye and Guo, 2019). A previous study of our group suggested that p73 independently predicted poor prognosis in colorectal cancer and p73-negative tumors tended to have a lower local recurrence after RT compared with unirradiated case (Ye and Guo, 2019).

One of important reasons is that the TP73 gene expresses isoforms with divergent and/or opposing roles in cancer. These are mainly categorized in two classes, the anti-oncogenic TAp73 isoforms, which contain an intact N-terminal, transactivation domain, and the oncogenic DNp73 isoforms, which lack part or whole of the transactivation domain and act as dominant negative forms of TAp73 proteins (Logotheti et al., 2013). The TAp73 isoforms are generated from an external P1 promoter. The DNp73 proteins are transcribed (a) by the P1 promoter, followed post-transcriptionally by alternative splicing in exons 2 and/or 3 at the 5′ end (Stiewe et al., 2002), or (b) by an alternative, internal P2 promoter which generates variants lacking exons 2 and 3, but instead containing an exon 3′ that encodes for a unique 13-amino acid domain (Irwin, 2006). Additional complexity is created by alternative splicing in the 3′ end, which gives rise to a large number of C-terminal variants of the abovementioned isoforms (Logotheti et al., 2013). Altogether, the TP73 gene expresses at least 35 mRNA variants, which can encode theoretically 29 different p73 protein isoforms (Murray-Zmijewski et al., 2006). Notably, the ratio between TAp73 and DNp73 isoforms has essential effects on the cellular response (Dulloo et al., 2010; Rufini et al., 2011).

The imbalance between TAp73 and DNp73 isoforms may be useful to predict response to chemotherapy and prognosis (Muller et al., 2005; Lucena-Araujo et al., 2015). High DNp73 expression has strong correlation with unfavorable prognosis in several types of cancer patients, and DNp73-positive tumors show a reduced response to chemotherapy and irradiation (Uramoto et al., 2004; Di et al., 2013; Zhu et al., 2015). The upregulation of DNp73 was frequently detected in radioresistant cervical cancers (Liu et al., 2006). Our previous findings indicated that DNp73 is increased in colon cancer cell line that is resistant to γ-irradiation (Pfeifer et al., 2009). Thus, these findings suggested that DNp73 expression may play an important role in the regulation of radiosensitivity. However, the prognostic and preditive role of DNp73 in rectal cancer patients with radiation still remains unclear.

This study aimed to elucidate the role of DNp73 as a predictive biomarker by investigating if DNp73 was related to the survival time of rectal cancer patients who were administered with RT before surgery. To overcome the subjective and time-consuming task of pathologist-based analysis of immunohistochemistry (IHC) images stained for DNp73 expression, we carried out a study by means of a novel image-based recurrence network approach. The motivation for developing this new image-based network analysis was based on the recurrence of image attributes inherently existing in the complex nature of IHC images of rectal cancer tissue arrays.

In fact, network analysis in graph theory has been increasingly recognized as a useful tool for studying cancer. Such studies include the prediction of outcomes of ovarian cancer treatment (Zhang et al., 2013), analysis of breast cancer progression and reversal (Parikh et al., 2014), drug response prediction in cancer cell lines (Zhang et al., 2018), identification of novel cancer gene candidates (Josef Gladitz et al., 2018), tumor biology for precision cancer medicine (Ozturk et al., 2018), and prediction of cancer recurrence (Ruan et al., 2019).

In this present study, we introduce a new method of fuzzy weighted recurrence networks of multi-channel images for computing useful properties of the complex networks of the expression patterns of the DNp73 IHC. The ratios of these network properties discover the predictive value of DNp73 in rectal cancer patients in the Swedish Rectal Cancer Trial.

## 2. Materials and Methods

### 2.1. Rectal Cancer Patients

This study included the patients with rectal adenocarcinoma from the Southeast Swedish Health Care region who participated in a clinical trial of preoperative RT for rectal cancer (Swedish Rectal Cancer Trial et al., 1997). Samples of biopsy and primary tumor from the same patients were selected for the analysis. In this Swedish Rectal Cancer Trial study, we collected samples from both pre-radiotherapy and non-radiotherapy rectal cancer patients. The biopsy samples were taken from the rectal cancer before the RT and went through the routine pathological process, and eventually embedded in paraffin blocks. The primary tumor samples were taken from the primary rectal cancer after the RT.

There were 25 patients with RT whose demographic information is given in Table 1. This study was carried out in accordance with the recommendations of Good Clinical Practice, the Research Ethics Committee in Linkoping, Sweden with written informed consent from all subjects. All subjects gave written informed consent in accordance with the Declaration of Helsinki. The protocol was approved by the Research Ethics Committee in Linkoping, Sweden. The clinico-pathologic characteristics of the patients are listed in Table 2.

**Table 1:** Demographic information of the rectal cancer patients who had a median age of 68 years (range: 39–78 years), were followed for a median period of 81 months (range: 0–129 months), and had the median time to disease free of 101 months after surgery (range: 15–288 months).

	**Number of patients**
Male	16 (64%)
Female	9 (36%)
Shorter survival time (15–75 months)	11 (44%)
Longer survival time (101–288 months)	14 (56%)

**Table 2:** Clinico-pathological characteristics of the rectal cancer patients.

**Parameters**		**Number of cases**
Age	<60	6
	>60	19
Gender	Male	9
	Female	16
Growth pattern	Expansion	11
	Infiltration	13
	Null	1
Grade	Well	2
	Moderate	14
	Poor	9
Pathological stages	I	8
	II	6
	III	8
	IV	3

### 2.2. Immunochemistry and Image Extraction

The five-micrometer paraffin-embedded tissue micro-array (TMA) sections were deparaffinized in xylene and rehydrated with a series of gradient ethanol to water. The sections were heated to boiling point in citrate buffer (pH 6.0) for 30 min to unmasked antigen, followed by a washing in phosphate-buffered saline (PBS). Endogenous peroxidase activity was blocked with 3% H_2_O_2_ in methanol followed by washing three-times in PBS. The sections were incubated with protein block (Dako, Carpinteria, CA) for 10 min and then incubated with anti-DNp73 antibody (clone 38C674.2, Novus Biologicals, 1:200), which specifically recognized DNp73 isoforms, but not TAp73.

After that, the sections were washed in PBS and then incubated with goat anti-mouse secondary antibody (Dako) at room temperature for 25 min. Next, the sections were subjected to 3,3′-diaminobenzidine tetrahydrochloride for 8 min and then counterstained with hematoxylin. Negative and positive controls were added in each staining run. All slides were scored by two independent investigators. Whole-slide images of entire sections were captured with an Aperio CS2 slide scanner system (Leica Biosystems, Wetzlar, Germany) using a 40x magnification.

All sections were reviewed to remove images containing tissue-processing artifacts, including bubbles, section folds and poor staining. A total of 46 whole-slide images from the 25 unique patients were extracted from the TMA slides using ObjectiveViewer (https://www.objectivepathology.com/objectiveview) with the original resolution.

### 2.3. Multi-Channel Fuzzy Weighted Recurrence Networks

The term “channel” is a conventional expression used to refer to a certain component of an image. For example, an RGB image has 3 channels that are red (R), green (G) and blue (B) components. A grayscale image has only one channel. Let **I** = [*f*_*ijk*_] be a multi-channel image of size *M* × *N* × *K*, where *i* = 1, …, *M*, *j* = 1, …, *N*, and *k* = 1, …, *K*. Let *m* ≥ 1 be an integer, a local image window ${\text{W}}_{ij}^{k}\in \text{I}$ of size (2*m*+1) × (2*m*+1) is constructed for each pixel located at *ij* in each of the *k* components of the multi-channel image, where *ij* is the center of the window. This window can be considered as embedding dimensions in two-dimensional space, which considers the local spatial distribution around *f*_*ij*_ of the *k*-th image channel. The Frobenius norm can be used to transform each local window into a scalar measure that has the useful property of invariance under rotations as

(1) $$\begin{array}{r} ||{\text{W}}_{ij}^{k}|{|}_{F}\;=\;\sqrt{\overset{i+m}{\underset{i-m}{\sum }}\overset{j+m}{\underset{j-m}{\sum }}|f_{ijk}{|}^{2}}, \end{array}$$

where (*i* − *m*), (*j* − *m*) > 0, (*i* + *m*) ≤ *M*, (*j* + *m*) ≤ *N*, and any pixel at the center of the window that requires values from beyond the image boundaries is skipped.

We can then obtain a set of feature vectors **y**_*ij*_, (*i*−*m*), (*j*−*m*) > 0, by joining the Frobenius norms computed for each window of the *k*-th image channel at the same location, for example, a color image of 3 channels:

(2) $$\begin{array}{r} {\text{y}}_{ij}\;=\;\left(||{\text{W}}_{ij}^{1}|{|}_{F},||{\text{W}}_{ij}^{2}|{|}_{F},||{\text{W}}_{ij}^{3}|{|}_{F}\right), \end{array}$$

where (*i* − *m*), (*j* − *m*) > 0, (*i* + *m*) ≤ *M*, (*j* + *m*) ≤ *N*.

Since the Frobenius norm induced feature vector set **y**_*ij*_ can be computed for the multi-channel image **I**, the multi-channel fuzzy weighted recurrence network (MC-FWRN), which is an extension of the FWRN of time series (Pham, 2019), can be constructed as follows. To simplify the notation in subsequent mathematical presentation, **y**_*ij*_ is now denoted as **x**_*n*_, *n* = 1, …, *L*, where *L* is the total number of feature vectors, and some same indices are used but defined differently.

Let **X** = {**x**_*n*_}, *n* = 1, …, *L*, *c* a given number of clusters of the feature space, and a set of *c* fuzzy clusters, **V** = {**v**_*i*_:*i* = 1, …, *c*}. Fuzzy clusters are groups that contain data points, where every data point has a degree of fuzzy membership of belonging to each group. A fuzzy relation $\tilde{\text{R}}$ between **v**_*i*_ and **v**_*j*_, *i, j* = 1, …, *c*, is characterized by a fuzzy membership function μ ∈ [0, 1], which expresses the degree of similarity of each pair (**v**_*i*_, **v**_*j*_) in $\tilde{\text{R}}$. This fuzzy relation has the following three properties (Zadeh, 1971):

1. Reflexivity: μ(**v**_*i*_, **v**_*i*_) = 1, ∀**v**_*i*_ ∈ **V**.
2. Symmetry: μ(**v**_*i*_, **x**_*n*_) = μ(**x**_*n*_, **v**_*i*_), ∀**x**_*n*_ ∈ **X**, ∀**v**_*i*_ ∈ **V**.
3. Transitivity: μ(**v**_*i*_, **v**_*j*_) = ∨_**x**_*n*__[μ(**v**_*i*_, **x**_*n*_) ∧ μ(**v**_*j*_, **x**_*n*_)], ∀**x**_*n*_ ∈ **X**, ∀**v**_*i*_, **v**_*j*_ ∈ **V**, where the symbols ∨ and ∧ stand for max and min, respectively.

The computation of μ(**v**_*i*_, **x**_*n*_), *i* = 1, …, *c*, *n* = 1, …, *L*, which are necessary for the construction of the fuzzy relation $\tilde{\text{R}}$ can be carried out by means of the fuzzy *c*-means (FCM) algorithm (Bezdek, 1981) as follows.

Let μ_*nj*_ denote a fuzzy membership grade of **x**_*n*_, *n* = 1, …, *L*, which belongs to a cluster *j*, *j* = 1, …, *c*, whose center is **v**_*j*_. This fuzzy membership is calculated by the FCM as

(3) $$\begin{array}{r} \mu_{nj}\;=\;\frac{1}{\sum_{i=1}^{c}{\left[\frac{d\left({\text{x}}_{n},{\text{v}}_{j}\right)}{d\left({\text{x}}_{n},{\text{v}}_{i}\right)}\right]}^{2/\left(\alpha -1\right)}}, \end{array}$$

where 1 ≤ α < ∞ is the weighting exponent, and *d*(**x**_*n*_, **v**_*j*_) is used as a Euclidean distance between **x**_*n*_ and **v**_*j*_.

Using the fuzzy membership grades, each cluster center **v**_*j*_ is computed as

(4) $$\begin{array}{r} {\text{v}}_{j}\;=\;\frac{\sum_{n=1}^{L}{\left(\mu_{nj}\right)}^{\alpha }\;{\text{x}}_{n}}{\sum_{n=1}^{L}{\left(\mu_{nj}\right)}^{\alpha }},\;\forall j. \end{array}$$

The iterative procedure of the FCM is outlined as follows.

1. Given *c*, α, step *t*, *t* = 0, …, *T*, initialize matrix ${\text{U}}^{\left(t=0\right)}\;=\;{\left[\mu_{nj}\right]}^{\left(t=0\right)}$
2. Compute ${\text{v}}_{j}^{\left(t\right)}$, *j* = 1, …, *c*, using Equation (4).
3. Update **U**^(*t*+1)^ using Equation (3).
4. If ||**U**^(*t*+1)^ − **U**^(*t*)^|| < ϵ or *t* = *T*, stop. Otherwise, set **U**^(*t*)^ = **U**^(*t*+1)^ and return to step 2.

The predefined FCM parameters α, *T* and ϵ usually take the values of 2, 100, and 0.00001, respectively. The number of clusters can be estimated using a cluster validity measure such as the partition entropy, denoted by *H*, which is defined as (Bezdek, 1981)

(5) $$\begin{array}{r} H\;=\;\frac{1}{L}\overset{c}{\underset{j=1}{\sum }}\overset{L}{\underset{n=1}{\sum }}\mu_{nj}\log\left(\mu_{nj}\right). \end{array}$$

This cluster validity works by computing the partition entropy *H* for a range of a given number of clusters, *c* ≥ 2, and the number of clusters that has the minimum value of *H* is considered as an optimal *c* for the FCM algorithm.

Finally, an *N* × *N* MC-FWRN can be constructed with the fuzzy relation $\tilde{\text{R}}$ as

(6) $$\begin{array}{r} \text{W}\;=\;\tilde{\text{R}}-\text{I}, \end{array}$$

where **W** is an *N* × *N* adjacency matrix of edge weights, and **I** is the *N* × *N* identity matrix. The interested reader is referred to the work described in Pham (2019) to obtain more detailed information about the concept of fuzzy weighted recurrence networks originally developed for time series.

### 2.4. Network Properties

Two most well-known measures of the statistical characterization of a complex network are the average clustering coefficient and characteristic path length (Watts and Strogatz, 1998; Albert and Barabasi, 2002; Barrat et al., 2004). The clustering coefficient of a node in a network is a numerical indicator of a node that tends to cluster with other neighboring nodes. The average clustering coefficient expresses the average amount of connectivity around individual nodes of a network, whereas the characteristic path length is considered as a measure of the efficiency of transfer of information in a network.

The average clustering coefficient for an unweighted network represented with an *N* × *N* (binary) adjacency matrix **A** = [*a*_*ij*_], *i, j* = 1, …, *N*, is defined as

(7) $$\begin{array}{r} C\;=\;\frac{1}{N}\overset{N}{\underset{i=1}{\sum }}C_{i}, \end{array}$$

where *C*_*i*_ is the local unweighted clustering coefficient for node *i*, and defined as

(8) $$\begin{array}{r} C_{i}\;=\;\frac{\sum_{j,k}a_{ij}a_{jk}a_{ki}}{k_{i}\left(k_{i}-1\right)},\;k_{i}\neq 0,1, \end{array}$$

where *k*_*i*_ is the degree of node *i*, which is the number of links of node *i*.

The average clustering coefficient for a weighted network is defined as

(9) $$\begin{array}{r} CC\;=\;\frac{1}{N}\overset{N}{\underset{i=1}{\sum }}C_{i}^{w}, \end{array}$$

where $C_{i}^{w}$ is the local weighted clustering coefficient for node *i*, and defined as (Fagiolo, 2007)

(10) $$\begin{array}{r} C_{i}^{w}\;=\;\frac{\sum_{j,k}\;{\left[w_{ij}w_{ik}w_{jk}\right]}^{1/3}}{k_{i}\left(k_{i}-1\right)},k_{i}\neq 0,1, \end{array}$$

where *w*_*ij*_, *w*_*ik*_, *w*_*jk*_ ∈ *W*.

In general, the clustering coefficient of a node is the ratio of existing links connecting a node's neighbors to each other to the maximum possible number of such links. The clustering coefficient for the entire network is the average of the clustering coefficients of all the nodes.

The characteristic path length of a network is defined as the average of all shortest path lengths:

(11) $$\begin{array}{r} CP\;=\;\frac{1}{N\left(N-1\right)}\overset{N}{\underset{i\neq j,i,j=1}{\sum }}d_{ij}, \end{array}$$

where *d*_*ij*_ is the length of the shortest path between nodes *i* and *j*. The Dijkstra's algorithm (Newman, 2010) was used for computing the shortest weighted path in this study. The characteristic path length is calculated by finding the shortest path between all pairs of nodes, adding them up, and then dividing by the total number of pairs. This operation shows on average the number of steps it takes to get from one node of the network to another.

### 2.5. Algorithm for Computing Network Properties From MC-FWRN

1. Given a multi-channel image **I**, window parameter *m*, number of clusters *c*, and FCM parameters.
2. Using Equation (1) to compute the Frobenius norm for each window (2*m* + 1) × (2*m* + 1) of each image channel, and using Equation (2) to form a matrix of vectors of length 3 with the number of pixels that can be used to construct the windows.
3. Compute the fuzzy weighted adjacency matrix **W** using Equation (6) via the FCM.
4. Using **W** to calculate the clustering coefficient with Equation (9), and the characteristic path length with Equation (11).

## 3. Results and Discussion

**Figure 1:**
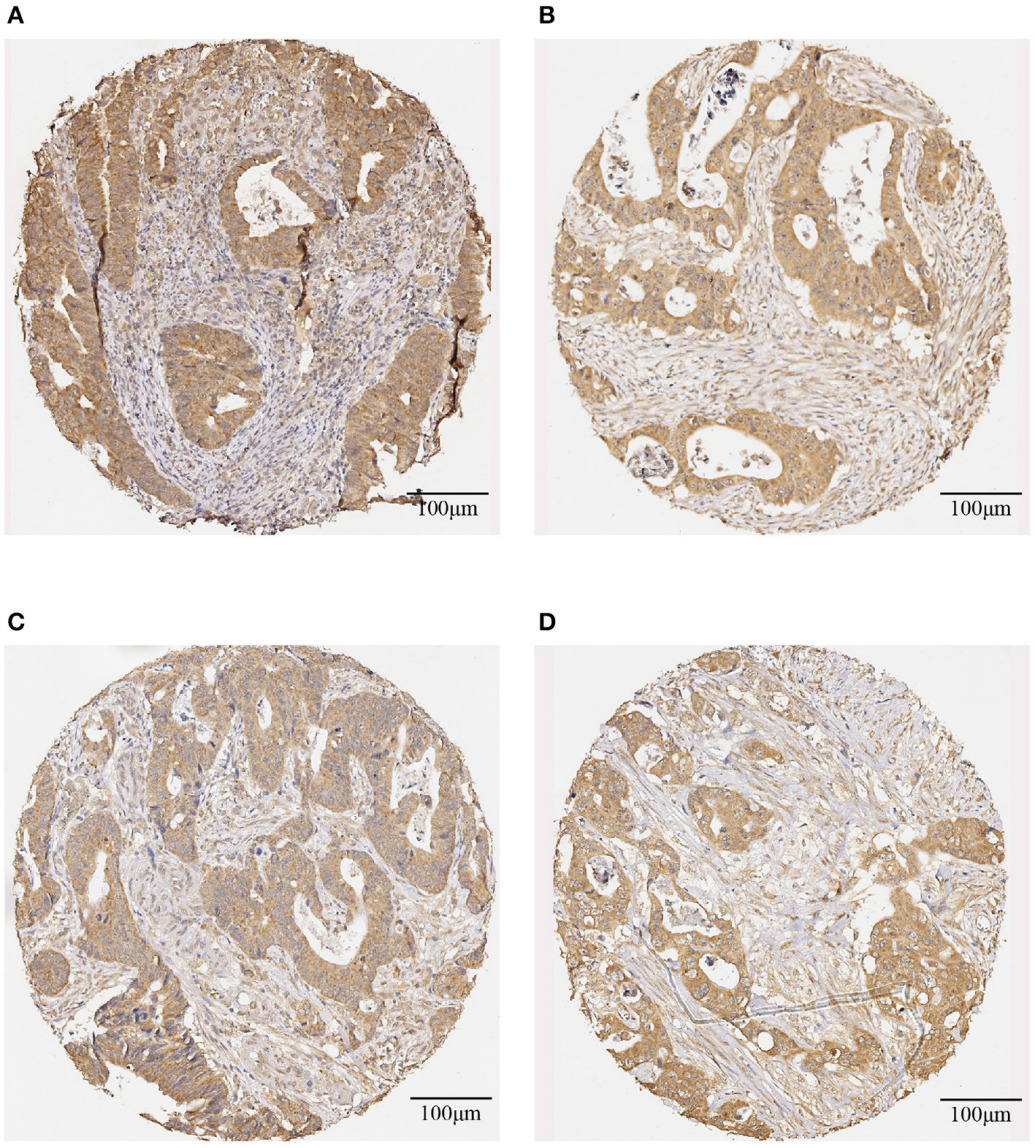
Representative IHC-stained images of DNp73 expression: **(A)** a biopsy image and **(B)** a primary tumor image obtained from a rectal cancer patient survived 40 months after radiotherapy; and **(C)** a biopsy image and **(D)** a primary tumor image obtained from a rectal cancer patient survived 255 months after radiotherapy.

Table 3 shows the screening results of the 25 rectal cancer patients. Patient numbers 1-11 are those who had shorter survival time, and patient numbers 12–25 are those who had longer survival time. The evaluation of the IHC-stained color intensity of the whole slide of a tissue core with brown antibody stain and blue counter-stain were assessed as being positive and negative, respectively. The positive stain is subjectively classified as weak = 1 (light brown), moderate = 2 (moderate brown), and strong = 3 (dark brown), whereas the negative stain = 0 (blue). Figure 1 shows representative IHC staining for DNp73 expression on the biopsy and primary tumor tissue images obtained from a rectal cancer patient survived 40 months after radiotherapy, and biopsy and primary tumor tissue images obtained from a rectal cancer patient who survived 255 months after radiotherapy at the censoring date.

**Table 3:** Screening results of rectal cancer patients.

**Patient #**	**Disease-free time**	**Recurrence status**	**Survival time**	**IHC score**	
				**Primary tumor**	**Biopsy**
1	0	Yes	15	1	1
2	6	Yes	19	1	3
3	20	Yes	25	2	1
4	13	Yes	40	3	3
5	37	Yes	60	3	3
6	44	Yes	62	2	3
7	0	Yes	15	3	2
8	63	Yes	75	3	3
9	26	Yes	27	3	3
10	12	No	26	3	3
11	34	Yes	43	2	2
12	100	Yes	101	1	1
13	0	Yes	180	1	3
14	114	Yes	114	2	2
15	122	Yes	255	3	2
16	167	Yes	167	2	2
17	81	No	101	3	2
18	129	Yes	129	2	3
19	129	Yes	288	2	3
20	126	Yes	126	3	2
21	186	Yes	238	3	3
22	122	Yes	122	2	2
23	168	Yes	288	2	3
24	151	Yes	151	3	3
25	168	Yes	168	2	2

*Time is in months. For IHC score, 0 = negative, 1 = weak, 2 = moderate, and 3 = strong*.

To capture the local information of the DNp73 expression over the whole IHC-stained slides, images of biopsy and primary tumor of each of the 25 rectal cancer patients were divided into subimages of 150 × 150 pixels. The subimages that contain either the background or a large portion of the background were excluded in the analysis. To construct the FWRNs of the IHC-stained subimages, we selected the FWRN parameters *m*=3 to establish a reasonable local window size of 7 × 7, *c* = 20 that was approximately based on the partition entropy, and the FCM parameters α = 2, *T* = 100, and ϵ = 0.00001, which are widely adopted for the FCM analysis. The clustering coefficient and characteristic path length were calculated for each subimage of each patient, and the total average values of the clustering coefficients and characteristic path lengths of all subimages represent the reported values.

**Figure 2:**
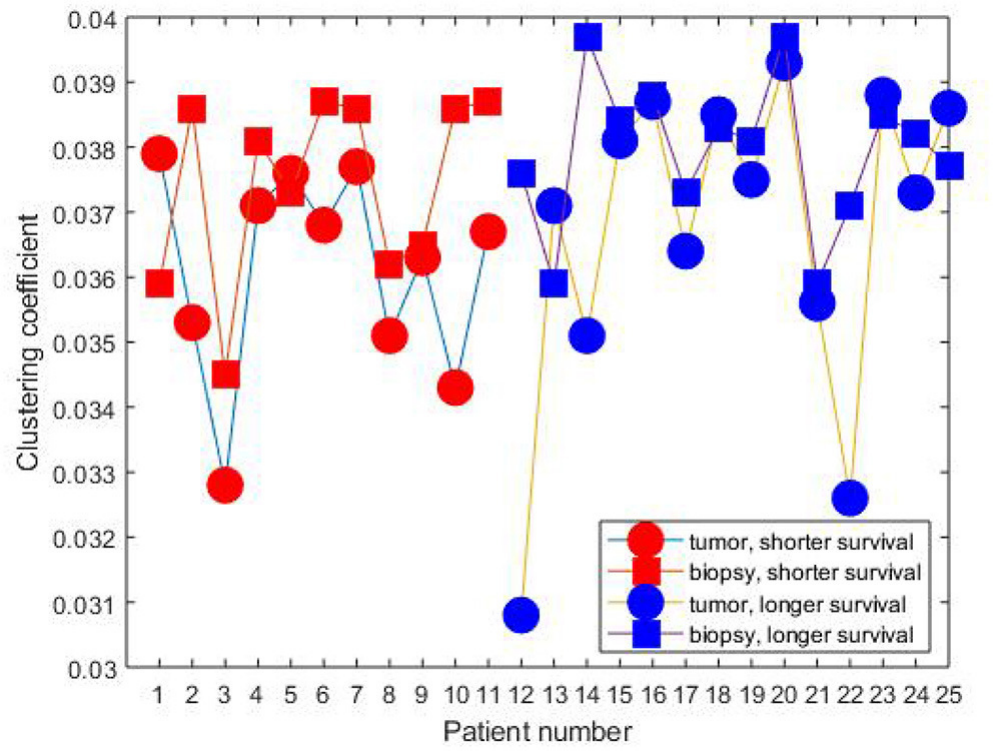
Clustering coefficients of rectal cancer patients.

**Figure 3:**
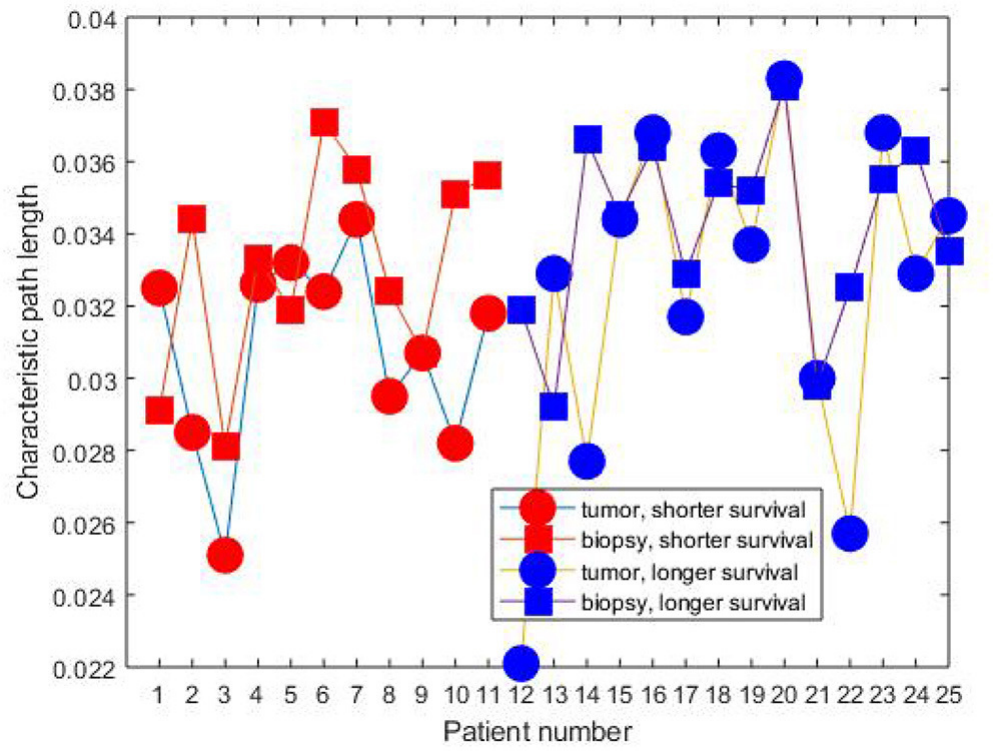
Characteristic path length of rectal cancer patients.

**Figure 4:**
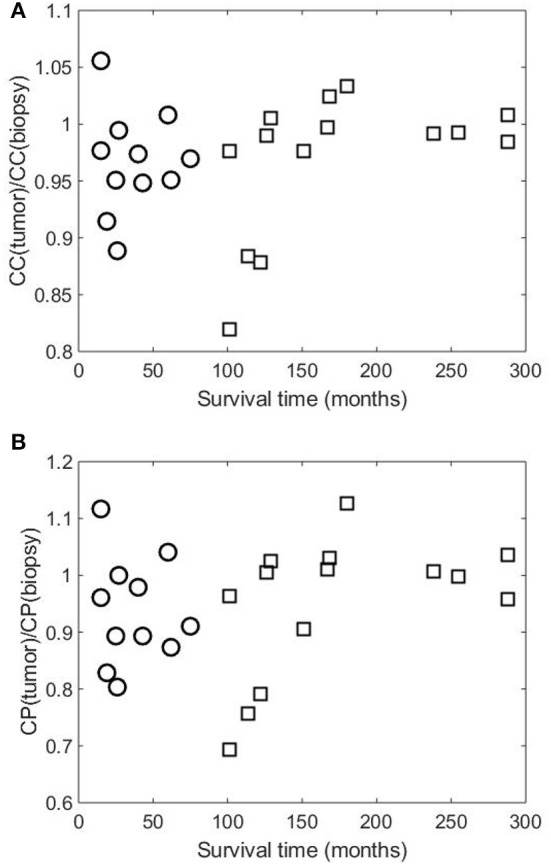
Scatter plots of survival time of 25 rectal cancer patient against **(A)** ratios of clustering coefficients of primary tumor [CC(tumor)] to those of biopsy [CC(biopsy)], and **(B)** ratios of characteristic path lengths of primary tumor [CP(tumor)] to those of biopsy [CP(biopsy)]. Symbols “○” and “□” indicate patient groups with shorter and longer times of survival, respectively.

Figures 2, 3 show the clustering coefficients and characteristic path lengths of the FWRNs of the biopsy and primary tumor images obtained from the 25 rectal cancer patients, respectively. The scatter plot of the survival time against the ratios of the clustering coefficients of the primary tumors to those of the biopsies, and the ratios of the characteristic path lengths of the primary tumors to those of the biopsies are shown in Figure 4.

**Figure 5:**
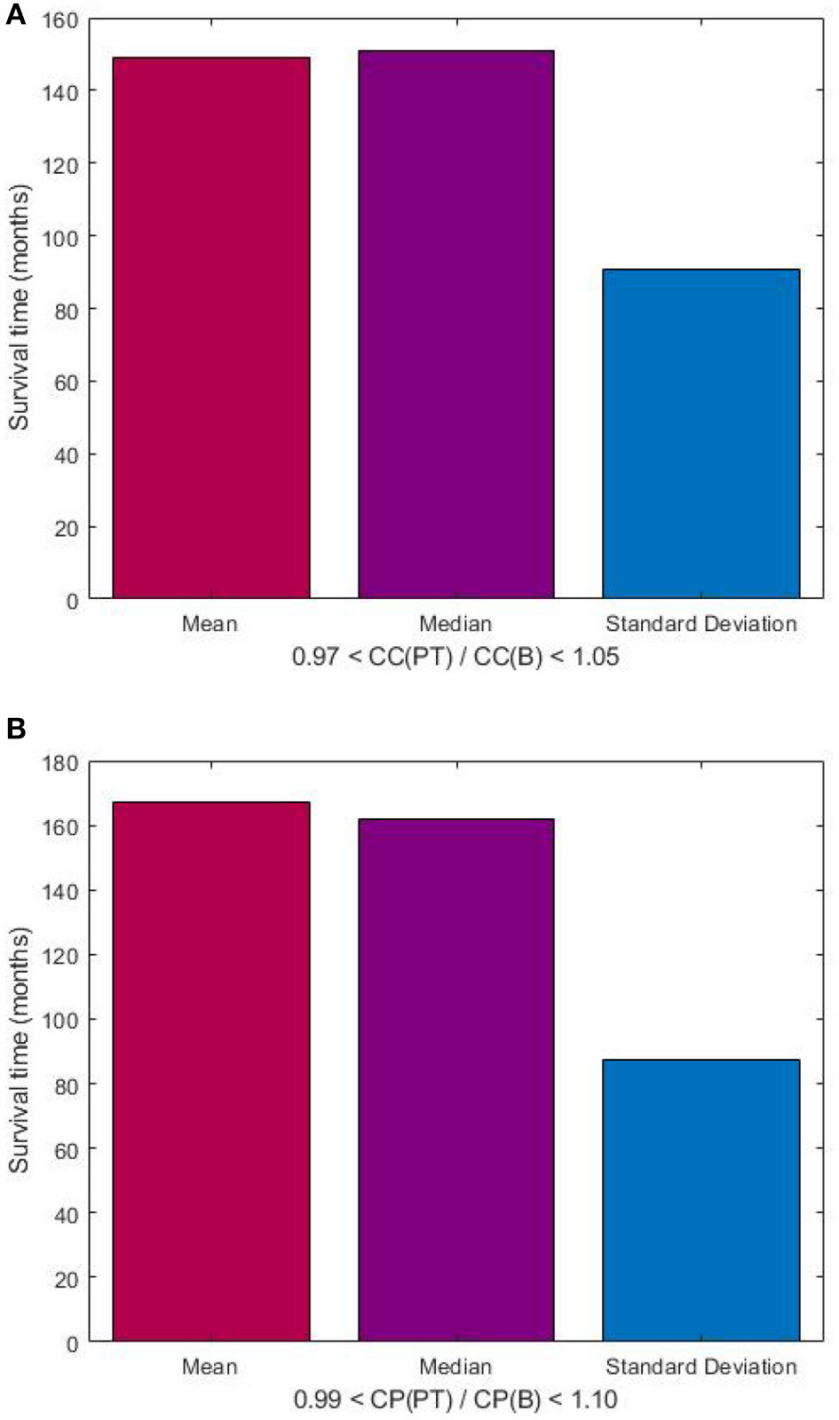
Predictive value of DNp73 in rectal cancer patients, who are deemed to have longer survival time as being between 101 and 288 months, in terms of ratios of clustering coefficient **(A)**, and characteristic path length **(B)** of image-based FWRNs. CC(PT) and CC(B) denote clustering coefficients of primary tumor and biopsy, respectively; and CP(PT) and CP(B) denote characteristic path lengths of primary tumor and biopsy, respectively.

Based on the visualization of the scatter plot, we discovered the predictive value of DNp73 in the rectal cancer patients in terms of the clustering-coefficient and characteristic-path-length ratios, which are shown in Figure 5. The probability (p) for the predicted survival time based on the clustering-coefficient ratio was computed as the number of patients who lived between 101 and 288 months divided by the total number of patients whose clustering-coefficient ratios are within the ratio range (*p* = 11/15 = 0.7333). The probability (p) for the predicted survival time based on the characteristic-path-length ratio was computed as the number of patients who lived between 126 and 288 months divided by the total number of patients whose characteristic-path-length ratios are within the ratio range (*p* = 7/9 = 0.7778).

Both intensity and percentage of the IHC staining have to be considered when we score the slides. We have been working with such a classic scoring system for many years. We have realized that even two experienced pathologists score the slides, there is still difficulty to make clear decisions for about 10% of the cases. In this study, a new image-based network analysis was developed to analyze the immunostaining array slides and to extract patterns of the IHC staining, including both intensity and percentage in the whole arrays. We further analyzed the associations of the immunostaining patterns with our clinical data to provide more precise information for rectal cancer.

The mean values of both clustering coefficients and characteristic path lengths of the rectal cancer patients of shorter survival are lower than those of longer survival. There is no correlation between the ratios of the clustering coefficients of the tumor to those of the biopsy and the survival time (correlation coefficient *R* = 0.0120, *p*-value = 9.7656e-04) among the shorter-surviving rectal cancer patients whose maximum survival time was about over 6 years (75 months). This can be observed from Figure 4. There is also no correlation between the ratios of the characteristic path lengths of the tumor to those of the biopsy and the survival time (*R* = −0.0780, *p*-value = 9.7656e-04) among the shorter-surviving patients. This can also be observed from Figure 4. There is an indication of correlation between the ratios of the clustering coefficients of the tumor to those of the biopsy and the survival time (*R* = 0.4924, *p*-value = 1.2207e-04) among the longer-surviving rectal patients whose maximum survival time was 24 years (288 months). This can be observed from Figure 4. There is also evidence of correlation between the ratios of the characteristic path lengths of the tumor to those of the biopsy and the survival time (*R* = 0.4778, *p*-value = 1.2207e-04) among the longer-surviving rectal cancer patients. This can also be observed from Figure 4.

Figure 4 shows similar plots of the ratios of the two network-property parameters of the tumor to biopsy against the survival time, suggesting the consistency of the results. It is reported that rectal patients who survive at least 5 years (60 months) are likely to die from causes that are common in the general population (London, 2017). This finding highlights the predictive value of DNp73 revealed by the image-based FWRN analysis among the cohort of rectal cancer patients whose survival time was between 8.4 years (101 months) and 24 years (288 months) correlated with the clustering-coefficient ratios, and 10.5 years (126 months) and 24 years (288 months) correlated with the characteristic-path-length ratios.

The lack of correlation of the ratios of the MC-FWRN parameters and the (shorter) survival time may suggest an implication of poor responses or non-effective treatment of the RT provided to the rectal cancer patients. Meanwhile, those patients who have positive correlation between the ratios of the FWRN parameters and the (longer) survival time were very likely to have a good or better response to the RT. General findings are that higher values of the ratios of the MC-FWRN parameters indicate longer survival time. The longest survival time is found with the values of the MC-FWRN parameter ratios being about 1. Based on the MC-FWRN parameters of the 25 rectal cancer patients and their survival months, we can predict the survival time between 101 months (8.42 years) and 288 months (24 years) with a probability of 73% for those patients whose clustering-coefficient ratio is within the range between 0.97 and 1.05. Similarly, the survival time between 126 months (10.5 years) and 288 months (24 years) with a probability of 78% for those patients whose characteristic-path-length ratio is within the range between 0.99 and 1.10.

The application of a novel image-based network analysis presented in this study was able to discover the predictive factor of DNp73 biomarker in rectal cancer patients having preoperative RT. Predictive biomarkers provide useful information on the probability of obtaining a response to treatment (Walther et al., 2009) and support the process of therapeutic decision for personalized cancer treatment (Voon and Kong, 2011). Such a discovery of DNp73 expression as a predictive biomarker in rectal cancer patients is expected to provide early assessment of the patient outcome, clinical value in the diagnostics of the disease, identification of targeted postoperative therapy.

Regarding the MC-FWRN introduced in this study, this new method appears to be the first of its kind mathematically formulated to capture the recurrence features of multi-channel data inherently existing in complex histology images in a way that is both effective and easily implemented for practical use. Complex networks consist of certain attributes that can be computed to analyze the properties and characteristics of the networks. Mathematical properties of these networks are utilized to define network models and to elucidate how certain models different to each other. The proposed MC-FWRN allows the calculation of the clustering coefficients and characteristic path lengths of DNp73 expression in the primary tumors and biopsies. These values can used to predict the survival time of a cohort of rectal cancer patients who were deemed to be positively influenced by preoperative RT.

The fuzzy weighted recurrence network analysis proposed herein is not supposed to be the study of the complexity of DNp73-controlled networks, but the derivation of structural properties of DNp73 expression from complex microscopy images that can be difficult to understand by pathologists. The results suggest that there are relationships between the graph properties of fuzzy weighted recurrence networks and the color distribution of the stained images. Hence, the network analysis yields new quantitative characteristics of the complexity of the IHC detection of the protein in tissue sections. From a molecular biology perspective, the average clustering coefficient and characteristic path length of the image-based fuzzy weighted recurrence network provide a mathematical measure of the heterogeneity of DNp73 in IHC staining, in correlation with clinicopathological characteristics. This heterogeneity may reflect diverse cell populations in expressing different levels of DNp73.

In this study, we have shown significant results concerning the DNp73 protein expression in predicting the outcome for the rectal cancer patients with the proposed mathematical approach. A limitation of this study is a relatively small number of the rectal cancer patients selected in the analysis. Therefore, future studies with more subgroups of rectal patients will be considered. It should be pointed out that although the total samples of the RT clinical trail from the Southeast Swedish Health Care region included 216 cases, only 102 cases randomly received preoperative RT. Given the aim of this study, only the paired samples of biopsy and primary tumors that are from the same patients were selected for the analysis. Many of the biopsy samples are too tiny to be used for IHC staining, constituting to the limitation of the sample size carried out in this pilot study, which still can provide some representative indication due to the paired samples from the same patient and all the samples derived from the random clinical trial.

Furthermore, results from rectal cancer patients with and without preoperative RT will be obtained and compared. Images of biopsies, primary cancers and metastatic cancers should be further investigated. Eventually, we will analyze the associations of the reactions from tumor invasive margins and stroma with the patients' prognosis.

Another limitation in this study is that the TAp73 expression was not performed in the present 25 pairs of rectal cancer samples. It is known that TAp73 acts as a tumor suppressor, while DNp73 exerts as an oncogene that is opposite to TAp73 (Amelio et al., 2014; Stantic et al., 2015). Therefore, it is necessary to expand the sample size and simultaneously evaluate TAp73 and DNp73 in the future, based on the methodology we have developed in the current study. DNp73 links to the ability to act as dominant-negative of the TAp73 isoforms and p53. This negative regulation by DNp73 forms an autoregulatory feedback loop, since both TAp73 and p53 can induce expression of DNp73 isoforms by direct binding to the P2 promoter (Irwin, 2006; Rufini et al., 2011; Di et al., 2013). A newest evidence showed that DNp73 isoforms has higher applicant potential in colorectal cancer patients than the canonical p73 protein (Garranzo-Asensio et al., 2019). Thus, it is reasonable to focus on DNp73. In addition, in the present paper, we mainly focused on the automated quantification of IHC expression using an image-based complex network model. The expression of TAp73 and the relationship between TAp73 and DNp73 will be investigated in our future study.

The highlights of the technical development and findings addressed in this paper are summarized as follows. First, the proposed MC-FWRN analysis of DNp73 expression by IHC in rectal cancer is the first of its kind. Second, a new mathematical analysis of IHC-stained biopsy and tumor images reveals the predictive power of DNp73 in rectal cancer patients who received RT. Third, a new method of multi-channel fuzzy weighted recurrence networks is developed for extracting two useful complex network properties of IHC images that can be used as prognostic indicators of rectal cancer. Fourth, the proposed approach for quantifying the expression of IHC is not limited to the study of DNp73, but can also be generally applied to discovering image patterns of other tumor proteins. Fifth, the proposed approach can be utilized as a computerized tool for extracting features from whole slide images in digital pathology.

## 4. Conclusion

The findings presented herein show the useful application of complex network analysis of images for studying the predictive factor of DNp73 biomarker expression in rectal cancer patients. The use of DNp73 biomarker can give insight into preoperative RT that has been considered as an important companion in the treatment of rectal cancer. A larger sample size when being available in future clinical trial will further confirm the current findings. Moreover, the proposed approach is not only found useful to rectal cancer but also can be adopted for the analysis of other biomarkers as well as other types of cancer, where human-based pathology practice is of limited capacity. In fact, there are many reports on the computerized image analysis of H&E (Haemotoxylin and Eosin) staining, much less effort has been made to apply computational methods for the automated analysis of IHC staining. The MC-FWRN presented in this paper can be generally applied for studying the expression of other potential biomarkers.

Although there are many studies reported about the association between DNp73 protein biomarker expression and malignant potential, the function of DNp73 still remains unclear. Our work contributes to the elucidation of the predictive value of DNp73 expression in rectal cancer patients who were given preoperative RT. We developed an original method for constructing weighted recurrence networks of multi-channel images. These networks allow the extraction of useful network properties from complex IHC images. The clustering coefficients and characteristic path lengths of the MC-FWRNs are not only able to show the predictive factor of DNp73 expression in the patients, but also reveal the identification of non-effective application of RT to those who had poor overall survival outcome.

## Ethics Statement

This study was carried out in accordance with the recommendations of Good Clinical Practice, the Research Ethics Committee in Linkoping, Sweden with written informed consent from all subjects. All subjects gave written informed consent in accordance with the Declaration of Helsinki. The protocol was approved by the the Research Ethics Committee in Linkoping, Sweden.

## Author Contributions

TP, HZ, and X-FS conceived the project. TP developed the fuzzy weighted recurrence networks of multi-channel images. TP, CF, HZ, and X-FS analyzed the results. TP, CF, and X-FS wrote the paper. TP carried out the computer implementation of the computational methods. DP contributed to the Lab work and data preparation. All authors edited and approved the manuscript.

### Conflict of Interest

The authors declare that the research was conducted in the absence of any commercial or financial relationships that could be construed as a potential conflict of interest.
